# Evidence mapping of preference elicitation for non-pharmaceutical interventions targeting respiratory viral transmission: A scoping review protocol

**DOI:** 10.1371/journal.pone.0344828

**Published:** 2026-05-29

**Authors:** Hui Yee Yeo, Ji Yeon Park, Samantha Marsh, Lorraine Castelino, Nikki Turner

**Affiliations:** Department of General Practice and Primary Care, University of Auckland, Auckland, New Zealand; ESIC Medical College and Hospital Faridabad, INDIA

## Abstract

**Introduction:**

Non-pharmaceutical interventions (NPIs) such as mask use, physical distancing, business or school closure, and isolation have been central to controlling respiratory viral infections, including influenza, COVID-19, and respiratory syncytial virus (RSV). Understanding the preferences of the public and stakeholders for these interventions is critical to ensure their acceptability, uptake, and effectiveness. Discrete choice experiments (DCEs) provide a robust method to quantify how individuals value different attributes of NPIs and the trade-offs they are willing to make. However, evidence from DCEs is fragmented across diseases, populations, and methodological approaches. This scoping review aims to systematically map and synthesise evidence from DCEs examining public and stakeholder preferences for NPIs used in the prevention and control of influenza, COVID-19, and RSV. Specifically, it will: (1) summarise the attributes and attribute levels used in DCEs, and the methods employed to select them; and (2) identify which NPI attributes are most valued by the public and key stakeholders, including patients, healthcare workers, and policymakers.

**Methods and analysis:**

The review will follow the PRISMA-ScR framework. Comprehensive searches of electronic databases (PubMed, Scopus, and Embase) will identify DCEs evaluating NPIs for influenza, COVID-19, and RSV. Data extraction will capture study characteristics, target populations, attributes, experimental design, and analytical methods. Reporting quality will be appraised using the DIRECT Checklist. Given anticipated heterogeneity, findings will be synthesised narratively. By providing a structured overview of existing DCE evidence, this review will inform the design of future preference studies and guide the development of acceptable and evidence-based NPI strategies for respiratory viral infections.

**Ethics and Dissemination:**

No ethical approval is required. The completed review will be shared through peer-reviewed journals and conference presentations.

Open Science Framework Registration https://doi.org/10.17605/OSF.IO/H36UC

## Introduction

Respiratory viral infections remain a major cause of morbidity, mortality, and socioeconomic disruption worldwide. Influenza has long posed a substantial seasonal burden, resulting in millions of severe cases and hundreds of thousands of deaths annually, causing huge economic and societal burden [1–3]. More recently, the coronavirus disease 2019 (COVID-19) pandemic highlighted the profound global consequences of emerging respiratory pathogens, adding unprecedented strain on healthcare systems and economies [4,5]. Respiratory syncytial virus (RSV), while often perceived as a paediatric illness, is increasingly recognised as a significant cause of severe disease among infants, older adults, and immunocompromised populations [6,7]. These three viruses share common modes of transmission, primarily via respiratory droplets, aerosols, and close contact, similar incubation periods, comparable seasonality patterns, as well as overlapping clinical manifestations, including fever, cough, and respiratory distress [8,9]. Consequently, their prevention and control strategies often rely on similar public health measures.

Public health responses to respiratory viruses consistently draw on a common set of non-pharmaceutical interventions (NPIs), including mask wearing, physical distancing, hand hygiene, testing and screening, isolation and quarantine policies, school and workplace closures, and travel restrictions. These measures constitute a cornerstone of respiratory infection control and work synergistically with vaccination and antiviral treatments to slow the spread of infection [10]. NPIs are also important in the early stage of a pandemic to delay spread and buy critical time for vaccine development. Guidance from the World Health Organization (WHO) explicitly outlines this shared NPI “toolbox” for pandemic preparedness and response, recommending similar measures across respiratory pathogens, including both influenza and COVID-19 [11,12]. During the COVID-19 pandemic, NPIs were rapidly implemented at an unprecedented scale, demonstrating their potential effectiveness in reducing transmission not only of SARS-CoV-2 but also of other respiratory viruses. For example, observational data from Hong Kong [13] showed that widespread adoption of NPIs for COVID-19 was associated with substantial reductions in both COVID-19 and influenza transmission. Systematic review evidence [14,15] similarly found that NPIs implemented during the COVID-19 pandemic led to marked declines in influenza activity and disruptions to the typical seasonality of RSV. In addition, nationwide register data from Finland [16] demonstrated that these interventions significantly suppressed RSV transmission.

Nevertheless, NPIs can impose social, psychological, and economic costs on individuals and communities, making their adoption and maintenance heavily dependent on public acceptance and stakeholder support [17]. The effectiveness of NPIs is therefore not determined solely by the specific pathogen targeted or their biological and epidemiological efficacy, but also by behavioural and social dynamics. Compliance with measures, such as mask use, isolation, or testing, is influenced by individuals’ perceptions of risk, trust in public authorities, perceived effectiveness, inconvenience, and the out-of-pocket costs associated with adherence [17,18]. From a theoretical perspective, these preference-related attributes are likely to operate in similar ways across attributes, regarding of the specific pathogen targeted [19,20]. This is because these attributes are inherent to the characteristics of the NPIs themselves rather than to disease-specific features. Therefore, policymakers and healthcare professionals must balance public health benefits against feasibility, equity, and ethical considerations when designing and implementing NPI policies. Failure to account for public and stakeholder preferences may undermine adherence and exacerbate inequalities, reducing the overall effectiveness of disease control strategies [21]. As such, understanding how different groups value attributes of various NPIs is essential for informing evidence-based, acceptable, and sustainable public health decision-making.

**Fig 1:**
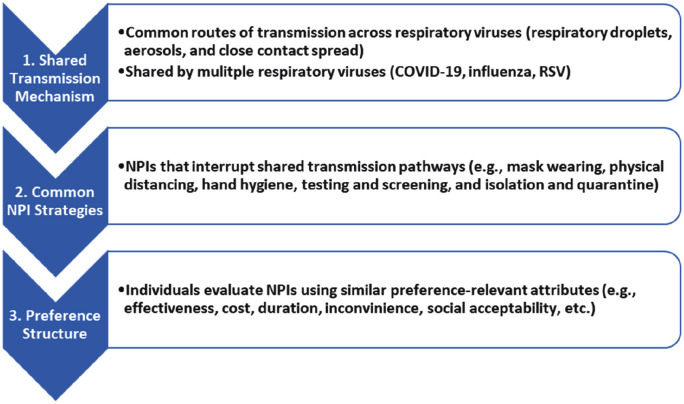
Conceptual framework: from transmission to preferences.

Fig 1 illustrates a three-stage conceptual framework linking transmission mechanism, NPI strategies, and preference structure. This framework illustrates how shared transmission mechanisms across respiratory viruses lead to common NPI strategies, which in turn generate similar preference structures, supporting the synthesis of public preference evidence for pandemic preparedness.

Preference elicitation methods have become increasingly important tools for capturing these perspectives in a systematic and quantitative manner. Among these, discrete choice experiments (DCEs) are widely used in health economics and public health to assess how individuals make trade-offs between different characteristics of healthcare services, interventions, or technologies [22,23]. Grounded in random utility theory, DCEs present respondents with hypothetical scenarios involving two or more alternative healthcare services, interventions, or technologies of interest. Each alternative is defined by a set of characteristics, known as attributes and levels, which allows researchers to estimate the relative importance of each attribute and individuals’ willingness to make trade-offs between them. Importantly, DCEs can be applied to diverse populations, including the general public, patients, healthcare workers, and policymakers, offering insights into preference heterogeneity across stakeholder groups.

The rapid proliferation of DCEs during and following the COVID-19 pandemic has generated a growing body of evidence on preferences for NPIs [24–27]. However, this literature remains fragmented across diseases, populations, and methodological approaches. Studies vary considerably in the attributes and attribute levels selected, the processes used to identify these attributes, and the analytical methods employed. Moreover, while COVID-19 has received substantial attention, preferences related to NPIs for influenza and RSV have been less comprehensively examined, despite the relevance of these findings for routine seasonal and future pandemic preparedness. This fragmentation poses challenges for synthesising evidence and translating findings into coherent policy recommendations.

A further limitation of the existing literature is the lack of a comprehensive synthesis that explicitly compares and integrates findings across respiratory viruses with similar transmission dynamics. Given the overlapping nature of NPIs used to control influenza, COVID-19, and RSV, there is a strong rationale for examining these diseases collectively rather than in isolation. Such an approach allows for the identification of common preference patterns, transferable insights, and context-specific differences that may inform more adaptable and resilient public health strategies. Including all three viruses also enhances the relevance of the review beyond emergency pandemic settings, extending its applicability to routine respiratory disease control and future outbreak preparedness.

### Aim

To conduct a scoping review that systematically synthesises evidence from DCEs examining public and stakeholder preferences for NPIs employed in the prevention and control of influenza, COVID-19, and RSV, and informs the development of a subsequent DCE investigating public preferences for public health measures involving NPIs during a flu pandemic.

### Specific objectives

1. To summarise the attributes and attribute levels used in DCE for measuring preferences for NPIs used in the prevention and control of influenza, COVID-19, and RSV, including the methods used for their selection.
2. To identify which attributes of NPIs are most valued from the perspectives of different stakeholders (e.g., public, patients, healthcare workers, policymakers).

## Methods

### Study design

The synthesis process and reporting of this scoping review will adhere to the methodological framework outlined in accordance with Arksey and O’Malley [28], Levac et al [29], and the PRISMA-ScR (Preferred Reporting Items for Systematic reviews and Meta-Analyses extension for Scoping Reviews) guideline [30]. We selected a scoping review as it is particularly well suited to addressing these types of broad, exploratory research questions and synthesising evidence from a body of literature that is methodologically and disciplinarily diverse [30]. Unlike traditional systematic reviews that focus on narrowly defined outcomes, scoping reviews aim to map the extent, range, and nature of existing evidence, identify key concepts and methodological approaches, and highlight gaps in the literature [28]. In the context of DCEs on NPIs, a scoping review can provide a structured overview of how preferences have been measured, which attributes have been prioritised, and whose perspectives have been captured. This information is critical for informing the design of future DCEs, improving methodological consistency, and supporting the integration of preference evidence into public health decision-making.

### Search strategy

A systematic search will be conducted via multiple databases: PubMed, Scopus, and Embase. Additionally, we will perform hand-searches of reference lists from included studies and relevant review papers identified during screening process to uncover additional studies for the review [31]. Each search will cover the period from 1990 until December 2025, as the first DCEs were published in the early 1990s in healthcare [32].

A subject matter expert librarian will guide the development of our search strategy, which will combine three key concepts: “influenza” OR “COVID-19” OR “respiratory syncytial virus” OR “other respiratory infections”, “NPI OR public health measures”, and “discrete choice experiment”. The three concepts will be combined using the “AND” operator. The search strategy will be initially developed in PubMed using Medical Subject Headings (MeSH) terms and keywords variants related to these concepts and will then be adapted for use across other databases.

For the NPI concept, we will consider those recommended by the WHO guideline to mitigate the risk and impact of epidemic and pandemic influenza [33] and COVID-19 [12]. The guideline specifically recommends measures in several domains: personal protective measures, environmental measures, social distancing measures, and travel-related measures. Furthermore, we will incorporate educational measures, contact tracing, and measures at entry points, such as screening procedures for individuals entering or leaving a country or region. A comprehensive list of the NPIs considered is provided in Table 1.

**Table 1:** The list of NPIs considered for this review.

NPI Measures	Sub-categories
Personal protective	Face masks
	Hand hygiene
	Gloves
	Cough or sneeze etiquette
Environmental	Surface or object sanitising/disinfecting
	Ventilation
	Humidity
Social distancing	Contact tracing
	Movement restriction/lockdown
	Isolation of sick individuals
	Quarantine of exposed individuals
	School measures and closure
	Workplace measures and closure
	Avoiding crowding
Travel-related	Entry and exit screening
	Internal travel restrictions
	Border closure
Education	Respiratory health literacy or education

### Search terms and keywords

Table 2 outlines the list of search terms and keywords considered in this review.

**Table 2:** Search terms and keywords.

Influenza
Influenza, human (MeSH)
Influenza*
Influenza A virus (MeSH)
Influenza B virus (MeSH)
H1N1 or PH1N1 or H3N2 or AH1N1 or AH3N2 or H5N1 or H7N9
Flu
**COVID-19**
COVID 19 or COVID-19 (MeSH)
Coronavirus
Corona virus
Coronal
2019-nCoV
SARS-CoV
SARS-CoV-2
Severe Acute Respiratory Syndrome coronavirus
MERS-CoV
Middle East Respiratory Syndrome coronavirus
**Respiratory Syncytial Virus**
Respiratory syncytial virus (MeSH)
Respiratory syncytial virus*
RSV
Respiratory infection (MeSH)
Respiratory infection*
Respiratory virus*
Influenza-like illness
ILI
Acute respiratory infection
ARI
Severe acute respiratory infection
SARI
**Other Respiratory Infections**
Respiratory infection
Respiratory viruses
Respiratory tract infections
**NPIs**
Non-pharmacological
Non-pharmaceutical
Face mask*
Personal protective equipment*/Personal protective gear*/Personal protective measure*
Glove*
Hand wash*/Hand hygiene
Disinfect*/Sanitisation/Sanitise/Sanitization/Sanitize
Surface clean*/Object clean*
School clos*
Travel restrict*/Movement restrict*/Travel advice/Travel advise
Quarantine
Social distanc*/ Physical distanc*
Avoid crowd*
Isolate/Isolation
Sick leave/ work from home/ working from home/ workplace clos*/ workplace absenteeism
Ventilation
Humidity
Respiratory Health literacy/ education/ training
Respiratory etiquette
Border clos*
Screen*/Scan*
Contact tracing
Public health measure*
Public health policy
Mitigation strateg*
Government response
Control
Prevention
**Discrete Choice Experiment**
Discrete choice experiment
Discrete choice
DCE
Choice modelling
Conjoint analysis
Conjoint
Best-worst scaling
Stated preference
Part-worth utility
Preference*
Choice*
Paired comparison
Pairwise

### Inclusion and exclusion criteria

Table 3 outlines the inclusion and exclusion criteria for this review. Only original articles published or accepted in peer-reviewed journals or reports from official public health bodies will be considered. All selected papers must be in English. The key distinction in our inclusion criteria is a focus on NPIs, that can be implemented without pharmaceutical agents. Diagnostic testing is not included because it is a tool that enables case identification and subsequent isolation decisions, rather than an intervention. Studies will be excluded if they do not explicitly examine the predefined NPIs listed in Table 1 or if they lack sufficient detail to clearly identify the methods, attributes, or attribute levels. Although travel‑related NPIs—such as travel restrictions, border closures, and quarantine requirements for travellers—will be included as public health measures targeting disease transmission, studies examining general transportation issues or travel mode preferences unrelated to infectious disease control will be excluded.

**Table 3:** Inclusion and exclusion criteria for the literature review.

	Inclusion	Exclusion
Context	Community-acquired human influenza, COVID-19, and RSV infection	Zoonotic infection Hospital-acquired respiratory infection
Population	General public, patients, healthcare workers, policymakers, and others	N/A
Intervention	Defined NPIs^#^	Vaccination; Antiviral strategy; other interventions not listed in the define NPIs list^#^
Outcomes	Preference for public health measures involving NPIs	Studies focused on general transportation or travel mode preferences not related to infectious disease control
Outcomes	Attributes and their associated levels used in DCE studies	Papers lacking adequate information to clearly delineate methods, attributes, or attribute levels
	Methods for attribute selection	
	Most preferred attributes	
Study Design	Discrete choice experiment Conjoint analysis Best-worst scaling	Purely qualitative studies
Article Type	Original articles published or accepted in peer-reviewed journals or reports	Letters, reviews, commentaries, editorials, case reports, case series, grey literature, and conference proceedings
Publication date	1990 – December 2025	N/A
Language	English only	N/A

# Defined NPIs: refer to Table 1 for the list of NPIs considered in this study.

We anticipate that the majority of included studies will be stated preference studies, particularly DCEs, given the inherent challenges in measuring revealed preferences for NPIs, which are often implemented at the population level and influenced by policy mandates and contextual constraints. However, we will not exclude revealed preference studies a priori. Any eligible studies that infer preferences from observed behaviours (e.g., uptake or adherence to NPIs) will be considered for inclusion, provided they meet the broader eligibility criteria. Where such studies are identified, they will be analysed and reported separately, with careful consideration of their methodological differences and limitations. This inclusive approach ensures that the review captures the full range of available evidence on preferences related to NPIs.

### Study selection

The studies identified through the search strategy will be exported to EndNote X9.1 and uploaded to the web application Rayyan (https://rayyan.ai/) [34] for screening. After removing duplicates, two reviewers (HYY and JYP) will independently screen the titles and abstracts of all retrieved studies based on predefined inclusion/exclusion criteria. The full texts of the studies that meet these criteria will then be independently reviewed by two reviewers (HYY and SM). Studies that do not meet the inclusion criteria will be excluded, with the reasons for exclusion documented in the final review. Any disagreements between the reviewers will be resolved through discussion, and if necessary, a third reviewer (JYP), will be consulted to make the final decision. Throughout the screening process, reviewers will maintain regular communication to accommodate the iterative approach of a scoping review, adjusting eligibility criteria as necessary. The study selection process will be presented using a PRISMA flow diagram.

### Data extraction and management

HYY will independently extract and consolidate data from each included study using a pre-defined and piloted data collection form designed specifically for this synthesis. The extracted data will be cross-checked by JYP and SM. Any discrepancies will be resolved by consensus among authors. Where necessary, the full author team will be consulted to reach agreement. Given the interpretive nature of some variables in this review, this approach is intended to ensure consistency and transparency in coding decisions while maintaining methodological rigor, without undertaking formal validation of inter-rater differences.

The initial data collection form will cover the elements shown in Table 4. The form will be piloted on a randomly selected sample of included studies (up to five, if available), after which the final version will be established. A modified version of the Coast et al. (2012) framework for the attribute development in DCE [35], which categorizes methods as: literature-based, qualitative research (interviews/focus groups), expert consultation, policy or guideline review, or combinations thereof, will be used to guide our categorization of attribute selection methods. We will also code whether attributes were developed de novo or adapted from existing instruments where possible.

**Table 4:** Data elements for abstraction.

	Domain	Data Extracted
1	First author	Name of first author
2	Publication year	Numerical number
3	Country	Country; not reported
4	Type of infection	Influenza; COVID-19; RSV
5	Type of NPIs	Open coding
6	Study population	Public; policy maker; healthcare professionals; others (specify)
7	Number of samples	Numerical number
8	Study design	DCE; conjoint analysis; best-worst scaling; others (specify)
9	Number of choice task	Numerical number
10	Choice task design: Labelled or unlabelled	Labelled; unlabelled
11	Choice task design: Forced choice or opt-out	Forced choice; opt-out
12	Study objective	Open coding
13	Methods used to identify attributes and levels	Literature-based; qualitative research (interviews/focus groups); expert consultation; policy/guideline review; combination; others (specify)
14	Hypothetical scenario/setting	Open coding
15	Number of attributes	Numerical number
16	Attributes and attribute levels used in each study	Open coding
17	Definition of each attribute	Open coding
18	Most valued/preferred attribute	Open coding
19	Utility models used	Open coding (e.g., conditional logit, mixed logit, hybrid modelling etc.)
20	Key findings	Open coding
21	Author’s conclusion	Open coding

### Reporting quality appraisal

The reporting quality of included studies will be appraised independently by HYY and JYP. Discrepancies will be solved by consensus and, if necessary, a third reviewer will be consulted (SM).

As no validated quality assessment framework currently exists for DCE in the health domain, the reporting quality of included studies will be appraised using reporting completeness as a proxy using the DIRECT Checklist [36]. In this review, the checklist will be applied to appraise key features of included studies, including the clarity of study objectives and rationale; the identification and specification of attributes and attribute levels; the experimental design of choice tasks and surveys; the appropriateness of econometric and statistical analyses; and the transparency of results reporting and interpretation.

To standardise the assessment process, each of the 18 items on the checklist will be evaluated using a three-level rating reflecting whether the criterion was not reported, partially reported, or fully reported. We will then summarise the findings descriptively by reporting (a) the percentage of studies addressing each DIRECT item, (b) patterns in items that are commonly reported versus those frequently omitted, and (c) a qualitative narrative describing overall trends in reporting quality. This descriptive approach avoids the false precision of generating an unvalidated composite score while still providing useful information about reporting practices.

### Ethics and dissemination

The final scoping review will be submitted to a peer-reviewed journal and may also be disseminated through relevant academic forums or conferences. Its findings will inform the development of a DCE examining public preferences for public health measures involving NPIs during a flu pandemic. This scoping review forms part of a larger DCE project, which has received approval from the Auckland Health Research Ethics Committee (LAH30293).

### Data synthesis and analysis

Given the expected substantial methodological heterogeneity across DCE studies, particularly in attribute selection and statistical analysis, direct quantitative comparisons (e.g., of regression coefficients) are not feasible. Rather than pooling heterogenous findings, results will be synthesized descriptively, with a focus on summarising the attributes and levels examined in each study to identify commonly valued aspects of non-pharmaceutical public health measures for influenza, COVID-19, and RSV. The narrative synthesis will be structured around key themes, including common versus unique attributes across studies, patterns in preference heterogeneity across population subgroups, the influence of methodological choices on reported outcomes, and relevant contextual factors such as temporal trends and geographic variation.

By drawing evidence from a variety of sources, this review will capture a comprehensive range of attributes related to NPIs and the preferences of different stakeholder, while acknowledging the limits of aggregation. The findings of this review will inform the development of attributes and levels for a future DCE aimed at evaluating the preferences and trade-offs of public health measures involving NPIs for future respiratory pandemic.

We will document and report the entire review process in accordance with the PRISMA-ScR checklist [30]. This will ensure transparency and comprehensiveness, covering all stages of the review including identification, screening, eligibility assessment, and inclusion of studies.

### Protocol amendments and deviations

Any amendments to the protocol will be systematically documented in a protocol amendment log, including the date, nature of the change, and the rationale. Minor clarification, such as typographical corrections or formatting adjustments, will be noted but will not require a formal protocol version update. In contrast, substantial changes (e.g., modification to inclusion/exclusion criteria, additions to data extraction table, changes to data synthesis and data analysis) will trigger a protocol version update and will be clearly reported in the final manuscript. We will also update our protocol registration on Open Science Framework register to reflect any amendments.

## Discussion

The overarching aim of this scoping review is to systematically map and synthesise the existing evidence on public preferences for non-pharmaceutical public health measures during pandemics, in order to identify key attributes, evidence gaps, and priorities to inform the design of a future DCE. This scoping review has several important strengths. First, this review adopts a broad and inclusive scope by examining DCEs related to NPIs across three major respiratory viral infections— influenza, COVID-19, and RSV. Given the similarities in transmission routes, clinical presentation, and mitigation strategies across these pathogens, this integrated approach allows for the identification of common patterns and transferable insights that extend beyond single-disease contexts.

Secondly, by focusing specifically on preference-based evidence derived from DCEs, this review provides critical insights into how different attributes of NPIs are valued and traded off by individuals and stakeholders. Unlike studies that examine preference, adherence or attitudes in isolation, DCEs allow for the quantification of preferences and the relative importance of intervention characteristics.

Thirdly, although scoping reviews do not typically require formal quality appraisal, this review incorporated a structured assessment of reporting quality using the DIRECT checklist. This additional step provides valuable context on the robustness and transparency of included studies and supports more informed interpretation of the evidence. Clear reporting enables readers to judge study quality and facilitates evidence synthesis by ensuring key methodological details are available. For a scoping review mapping the landscape of DCE evidence, documenting reporting practices is itself an important outcome.

Finally, because the findings of this scoping review will directly inform the development of a comprehensive DCE, supplemented by qualitative interviews and a complementary review of policy documents across multiple countries, it was essential to capture the full range of factors influencing preferences for, and the acceptability and uptake of NPIs. Incorporating these factors will support the optimal design of the subsequent DCE.

Despite these strengths, several limitations should be acknowledged. First, considerable methodological heterogeneity is anticipated across the included DCEs, particularly regarding attribute selection and analytical approaches. Furthermore, as a scoping review, this study aimed to map and describe the existing evidence rather than to assess effectiveness or establish causal relationships. Consequently, the review does not provide direct quantitative comparisons, pooled estimates or definitive conclusions regarding optimal NPI design, but instead offers a structured narrative overview to inform future research and policy development.

Second, the review may be subject to publication and language bias, as only studies published in peer-reviewed journals and in English were included. Relevant grey literature or unpublished studies, as well as studies published in other languages, may therefore have been missed. Furthermore, since the DIRECT checklist only assesses reporting completeness, a well-reported study may still have methodological limitations. Therefore, the reporting completeness appraisal should not be interpreted as definitive evidence of study quality, and that the readers of this review should critically appraise individual studies based on their specific research questions.

Finally, preferences elicited through DCEs are inherently context-specific and may be influenced by the timing of data collection, particularly in rapidly evolving situations such as the COVID-19 pandemic. Therefore, the findings may not be fully generalisable across settings, populations, or stages of an outbreak. To address this limitation, we will carefully document the context in which each study was conducted, including the population characteristics, timing relative to epidemic waves, and prevailing public health measures.

## Supporting information

S1 File Appendix A. The search strategy utilised for PubMEd using Medical Subject Headings (MeSH) terms and keywords variants related to these concepts.
(DOCX)

S2 File PRISM-ScR Checklist. Preferred Reporting Items for Systematic reviews and Meta-Analyses extension for Scoping Reviews (PRISMA-ScR) Checklist.
(PDF)
