# Supplementary material for: Evidence mapping of preference elicitation for non-pharmaceutical interventions targeting respiratory viral transmission: A scoping review protocol
Source: PLoS One. 2026 May 29;21(5):e0344828. doi: 10.1371/journal.pone.0344828 (PMC13221047; doi:10.1371/journal.pone.0344828)
Supplement: S1 File — The search strategy utilised for PubMEd using Medical Subject Headings (MeSH) terms and keywords variants related to these concepts. (DOCX) [file pone.0344828.s001.docx]

**Search Strategy in PubMed**

| Search | Query |
| --- | --- |
| #1 | (((influenza, human[MeSH Terms]) OR (influenza[Title/Abstract] OR flu[Title/Abstract])) OR (("Influenza A virus"[MeSH Terms]) OR ("Influenza B virus"[MeSH Terms]))) OR (H1N1[Title/Abstract] OR PH1N1[Title/Abstract] OR H3N2[Title/Abstract] OR AH1N1[Title/Abstract] OR AH3N2[Title/Abstract] OR H5N1[Title/Abstract] OR H7N9[Title/Abstract]) |
| #2 | ((((((covid-19[MeSH Terms]) OR ("covid 19"[MeSH Terms])) OR (coronavirus[MeSH Terms])) OR ("severe acute respiratory syndrome related coronavirus"[MeSH Terms])) OR ("sars cov 2"[MeSH Terms])) OR ("Middle East Respiratory Syndrome coronavirus"[MeSH Terms])) OR (COVID-19[Title/Abstract] OR "COVID 19"[Title/Abstract] OR Coronavirus[Title/Abstract] OR "Corona virus"[Title/Abstract] OR Coronal[Title/Abstract] OR 2019-nCoV[Title/Abstract] OR SARS-CoV[Title/Abstract] OR SARS-CoV-2[Title/Abstract] OR MERS-CoV[Title/Abstract] OR "Middle East Respiratory Syndrome coronavirus"[Title/Abstract]) |
| #3 | ("Respiratory syncytial viruses"[MeSH Terms]) OR ("Respiratory syncytial virus"[Title/Abstract] OR "Respiratory syncytial viruses"[Title/Abstract] OR RSV[Title/Abstract]) |
| #4 | ("respiratory tract infections"[MeSH Terms]) OR ("Respiratory infection"[Title/Abstract] OR "Respiratory virus"[Title/Abstract] OR "Influenza-like illness"[Title/Abstract] OR ILI[Title/Abstract] OR "Acute respiratory infection"[Title/Abstract] OR ARI[Title/Abstract] OR "Severe acute respiratory infection"[Title/Abstract] OR SARI[Title/Abstract] OR "respiratory infections"[Title/Abstract] OR "Respiratory viruses"[Title/Abstract]) |
| #5 | (#1 OR #2 OR #3 OR #4) |
| #6 | Non-pharmacological[Title/Abstract] OR Non-pharmaceutical[Title/Abstract] OR "Face mask"[Title/Abstract] OR "face masks"[Title/Abstract] OR "Personal protective equipment"[Title/Abstract] OR "Personal protective gear"[Title/Abstract] OR "Personal protective measure"[Title/Abstract] OR "Personal protective measures"[Title/Abstract] OR Glove*[Title/Abstract] OR "Hand wash"[Title/Abstract] OR "Hand hygiene"[Title/Abstract] OR Disinfect*[Title/Abstract] OR Sanitisation[Title/Abstract] OR Sanitise[Title/Abstract] OR Sanitization[Title/Abstract] OR Sanitize[Title/Abstract] OR "Surface clean*"[Title/Abstract] OR "Object clean*"[Title/Abstract] OR "School clos*"[Title/Abstract] OR "Travel restrict*"[Title/Abstract] OR "Movement restrict*"[Title/Abstract] OR Quarantine[Title/Abstract] OR "Social distanc*"[Title/Abstract] OR "Physical distanc*"[Title/Abstract] OR "Avoid crowd*"[Title/Abstract] OR Isolate[Title/Abstract] OR Isolation[Title/Abstract] OR "Sick leave"[Title/Abstract] OR "work from home"[Title/Abstract] OR "working from home"[Title/Abstract] OR "workplace clos*"[Title/Abstract] OR "workplace absenteeism"[Title/Abstract] OR Ventilation[Title/Abstract] OR Humidity[Title/Abstract] OR "Respiratory Health literacy"[Title/Abstract] OR "Respiratory Health education"[Title/Abstract] OR "Respiratory Health training"[Title/Abstract] OR "Respiratory etiquette"[Title/Abstract] OR "Border clos*"[Title/Abstract] OR Screen*[Title/Abstract] OR Scan*[Title/Abstract] OR "Contact tracing"[Title/Abstract] |
| #7 | "discrete choice experiment"[Title/Abstract] OR "discrete choice"[Title/Abstract] OR DCE[Title/Abstract] OR "Choice modelling"[Title/Abstract] OR "conjoint analysis"[Title/Abstract] OR conjoint[Title/Abstract] OR "Best-worst scaling"[Title/Abstract] OR "Stated preference"[Title/Abstract] OR "Part-worth utility"[Title/Abstract] OR Preference*[Title/Abstract] OR Choice*[Title/Abstract] OR "Paired comparison"[Title/Abstract] OR pairwise[Title/Abstract] OR trade-off*[Title/Abstract] OR "trade off*"[Title/Abstract] |
| #8 | (#5 AND #6 AND #7) |

Notes:

Our search strategy employs: controlled vocabulary (MeSH terms in PubMed and Emtree in Embase) combined with free-text searching, truncation symbols to capture word variants (e.g., mask* to capture mask, masks, masking), and comprehensive synonym lists for all key concepts. Boolean operators will use ‘OR’ to combine terms within concept groups and ‘AND’ to link the groups.
